# The Probable Origin of the Red Blood-Corpuscles

**Published:** 1894-12

**Authors:** 


### The Probable Origin of the Red Blood-Corpuscles.

The supply of red blood-corpuscles is apparently maintained from the nucleated red blood-cells of the bone marrow, the structure of which is described by Dr. Robert Muir and Dr. W. B. Drummond in the *Journal of Anatomy and Physiology.* In the marrow of man and other mammals the supporting stroma is very slender, and at the periphery it is arranged in one or two layers of connective-tissue corpuscles with fibrils between. From these layers delicate nucleated filaments press inward and are connected with the blood-vessels and fat cells. There are usually in the center of the marrow a single artery and a much larger vein. The branches of the artery terminate in what may be called arterial capillaries, and these empty into rather wide channels which may be termed venous capillaries, though they do not have a complete endothelial lining. The bulk of the bone marrow is composed of spherical, colorless, granulated marrow cells. There are also many nucleated cells containing hemoglobin, which are larger than red blood-corpuscles, and are termed erythroblasts. Giant cells are found with a single lobulated nucleus appearing as many in one focus of the microscope. As the parenchyma is nearly cut off from the venous blood, the circulation through the marrow must be slow. Dr. W. H. Howell, of Johns Hopkins University, maintains that the marrow cells are embryonic cells whose function is to multiply by division and become nucleated red blood-cells. During their successive divisions the cells become smaller and pass from the type of the marrow cell to that of the nucleated red blood-corpuscle. But Dr. Muir considers that the marrow cells are of the leucocyte order. The red corpuscles found in the marrow substance have probably just lost their nuclei. Nucleated red corpuscles are not found in normal blood, but after severe hemorrhage in the human subject and in animals they may appear in the general circulation, in which case they stain more deeply with methyl blue than the ordinary red blood-corpuscle. Dr. Muir concludes that the nucleated red cells of the bone marrow are probably in free communication with the blood stream, but normally do not enter it, being retained in position by mutual cohesiveness, even after they have lost their nuclei. In this position they gradually acquire the physical properties of the adult red blood-corpuscles, and then pass into the flowing current. He reasons that after severe hemorrhage there occurs a dilution of the blood, in respect to the red corpuscles, and therefore the corpuscles are loosened in the vascular channels of the bone marrow where they are normally closely packed, and thus the normally stationary red cells may pass into the general circulation. The richness of the blood in corpuscles may be looked upon as determining the stability of the cellular arrangement in the marrow. Poverty in blood-corpuscles may not only induce certain cells to enter the circulation, but also stimulate other cells to proliferate, as it is thoroughly well established that the multiplying cells become smaller and pass from the type of the marrow cells to that of the nucleated red blood-cell, which so closely resembles the red blood-corpuscles.—*N. Y. Med. Journal.*

